# Implementing team science in undergraduate medical physics research

**DOI:** 10.1002/acm2.70169

**Published:** 2025-07-14

**Authors:** Ashley J. Cetnar

**Affiliations:** ^1^ Department of Radiation Oncology The Ohio State University Columbus Ohio USA

**Keywords:** education, team science, undergraduate research

## Abstract

**Purpose:**

Many undergraduate students are eager to learn more about potential career opportunities. While physics majors are often aware of research opportunities within their home department, students may not be aware of how physics can be applied in medicine. An alternate framework for a 10‐week summer undergraduate research experience using a collaborative Team Science approach is presented.

**Methods:**

The Team Science program is described, with feasibility shared based on the experiences of four students who piloted the program. Students explored four different projects throughout the summer as part of the 10‐week program and assumed different team roles for each project fostering experience understanding team dynamics. Changes in student attitudes toward science research were quantified using validated surveys and qualitative responses are also summarized.

**Results:**

Average self‐reported student scores in the Attitudes and Approaches to Problem Solving Survey increased 9.8% and Colorado Learning Attitudes about Science Survey for Experimental Physics increased 14.2% after the summer research experience. Areas with the highest reported gains from the program from the Undergraduate Research Student Self‐Assessment Survey included understanding what everyday research work is like, engaging in real‐world science research, and preparation for the future.

**Conclusions:**

Using a Team Science approach allowed for students to explore multiple research questions during the summer and experience a more authentic and integrated approach to how research is conducted. This model can be expanded and adapted for students to identify medical physics as a career for applying their physics knowledge to solve problems that will advance human health earlier in their career.

## 1 INTRODUCTION

Unlike the popular notion that scientists work alone in a laboratory, science is collaborative and social. Medical physics combines traditional disciplines such as physics, biology, medicine, and engineering to solve novel problems. Undergraduate research experiences are pivotal opportunities for students to experience what is like to be a scientist beyond the classroom.^1^, ^2^ Existing programs are sponsored by the American Association of Physics in Medicine (AAPM) which include the Summer Undergraduate Fellowship Program and the Diversity Recruitment through Education and Mentoring Programs,^3^, ^4^, ^5^ and other institutions also sponsor undergraduate research for medical physics.^6^, ^7^, ^8^

While programs for undergraduate research experiences have demonstrated value of providing opportunities for students and recruitment to the field, the format for the program is typically designed as individual mentoring for 8–10 weeks during the summer. To provide an authentic experience of what it is like to be a medical physicist, we piloted a unique model based on a collaborative Team Science^9^ approach proposed by the National Cancer Institute (NCI) applied for undergraduate physics students. The aim of this study is to provide medical physicists with an alternate framework for mentoring research experiences for undergraduate students.

Learning objectives of this program were to:

1. inspire students willing to explore new topics and a career path in medical physics
2. extend a welcoming environment to encourage students to be a part of the community
3. provide students an opportunity to participate in research with both breadth and rigor
4. and offer an opportunity for students to take on various responsibilities and leadership within a team.

## 2 INNOVATION

### 2.1 Team science

While most undergraduate research programs attempt to absorb individuals into existing research teams, this traditional method has had varying success in outcomes in terms of applying knowledge and technical skills, increasing retention in STEM disciplines, and workforce development depending on factors such as the individual learner, culture of the existing group, mentorship, and program structure.^2^, ^10^, ^11^ The transition from an individual approach to research for this project was motivated by the challenge that “…*society's problems do not fit neatly into the University's departmental grid, nor are they rapidly divisible into subproblems… interdisciplinary research teams can readily respond to multi‐discipline, problem‐oriented research and public service opportunities*.”^12^

“Team Science” for this program is defined as a collaborative effort in which multiple people come together to solve complex problems that would not be solved as effectively as individuals working on their own. Benefits of Team Science have been shown to provide opportunities to acquire knowledge faster, foster creativity, develop multiple skills, and address complex problems in challenging environments.^13^, ^14^, ^15^ Within scientific fields, evidence has shown that transdisciplinary teams can produce more highly cited research than individuals in the long term, indicating a mix of diverse perspectives and approaches can produce impactful work.^16^, ^17^, ^18^ The importance of collaboration among scientists has been shown by number of publications, serving as a proxy for academic productivity.^19^, ^20^, ^21^

### 2.2 Program structure

The goal of the medical physics research experience was to create an opportunity for undergraduate students to have an authentic experience of what it is like to be a modern scientist. The program was designed to provide a structured mentoring environment where learners could investigate multiple projects for learners to solve real‐world problems in medicine by applying knowledge and understanding physics, biology, engineering, mathematics, and computer science. Structure of the program was based on the NCI Collaboration Team Science Field Guide to convey overall goals and vision, project management, and participant communication.^9^, ^22^, ^23^, ^24^

This program was designed to allow undergraduates to work together to conduct research and have peer support throughout the process instead of pursuing a single research question in isolation. By working in groups, individuals gained experience learning how to function effectively in teams, strengthening communication skills, and improving overall attitudes toward science. Within the pilot program, four undergraduate physics students worked collaboratively to solve assigned problems within the team. This program was non‐traditional in the sense that it was not structured to provide a 10‐week experience focused on a single topic and strategically promoted collaboration through Team Science.

Teams had the opportunity to develop and test several hypothesis‐driven research questions throughout the program, so students were not compartmentalized into the focus of one specific topic for their summer experience. The students solved different problems with the goal of sparking interest in an area for further pursuit of medical physics beyond the program. The objective for the program director was to design each of the problems to be well defined and feasible for the teams to complete within the 10‐week period. In this pilot program the students collaborated to complete four distinct 2‐week projects throughout the summer. An example of the structured progression through each research question cycle is shown in Table 1.

**TABLE 1:** Example progression for each hypothesis‐driven research question cycle.

Week A				
Monday	Tuesday	Wednesday	Thursday	Friday
Project introduced	Research plan development	Gathering information	Experimental design	Experimental design
Team brainstorming and question development	Formal meeting with project mentor		Formal meeting with project mentor	Discussion and reflection

Week B				
Monday	Tuesday	Wednesday	Thursday	Friday
Conducting experiment	Conducting experiment	Synthesizing data	Writing results	Present results
	Formal meeting with project mentor		Formal meeting with project mentor	Discussion and reflection

### 2.3 Models of collaboration

The participants in the summer research experience were coached in collaborative team dynamics and provided opportunities for reflection, while the program director guided them through stages of collaboration to support their progress throughout the program. Frey et al. summarized phases of team collaboration across a spectrum representing collaboration models from five theorists which are anchored by the Seven Stage Model: coexistence, communication, cooperation, coordination, coalition, collaboration, and coadunation.^25^ The students started at the stage of coexistence at the beginning of the program. After the first week's orientation, the team transitioned to the next level in this model which focused on effective communication and cooperation was promoted by introducing activities that required teamwork. During the first projects, the learners were expected to continue to communicate and cooperate towards their group and individual goals, but also began aspects of coordination, coalition, and collaboration which developed throughout the program.

Participants were introduced to various aspects of Team Science during the introduction week including self‐reflection, assessment, case studies. In the first week of orientation, the program director sought to understand the strengths of the learners through the Clifton StrengthsFinder^26^ as a tool to develop the team by identifying top strength areas for each individual in terms of strategic thinking, relationship building, influencing, and executing identified by 34 distinct themes.

The program director designed the project questions based on the experience and interest of the students with the available institutional resources. Each research question was posed at the beginning of the 2‐week cycle by the director and then was explored by the team with guidance of the director and mentors to develop skills and access resources to answer the research questions.

**FIGURE 1:**
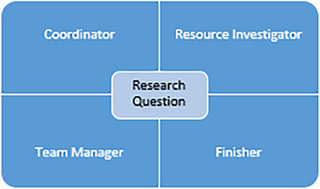
Assigned roles within team for each research question. The individual roles rotated throughout the program.

Each learner was assigned a primary role within the team for each research question including the “Coordinator” responsible for chairing the project and overall logistics, “Resource Investigator” exploring options and opportunities, “Team Manager” encouraging cooperation between team members, and “Finisher” ensuring timelines are met and work is thorough (Figure 1). These roles rotated for each project cycle, so learners were able to experience different perspectives and responsibilities within the team. Other aspects of the program beyond the specific research questions included clinical shadowing, workshops for skill building (i.e. Monte Carlo modelling), and professional networking lunches to have a holistic understanding of the scope of what it means to be a medical physicist.

## 3 COMMUNICATION

Communication of participants within the program was overseen by the program director. During initial orientation, learners had the opportunity to meet one another and faculty leading the projects. Learner‐learner communication was established through formal group meetings. The student's work area was configured as an office of four workspaces to support daily team interactions. Collaboration tools were encouraged including the use of digital lab notebooks, collaboration and communication platforms, and virtual tackboards to explore options for team management. The students were encouraged to meet outside of research times for informal social events for relationship building. Networking lunches were scheduled to help promote a venue for networking among the participants and medical physicists. Medical physicists and residents specializing in radiation oncology and diagnostic imaging used an online sign‐up for daily lunches to meet with the students providing a venue to discuss professional topics, build relationships, and ask questions about the field.

## 4 PROGRAM EVALUATION

Assessments can help educators identify and understand factors that influence student engagement and interest in a topic. Attitude assessments are commonly used in physics education research to improve learning environments in a way that is data driven to enhance the overall educational experience. Several validated assessments have been developed to better interpret undergraduate student research experiences.

### 4.1 Attitudes and Approaches to Problem Solving Survey

The Attitudes and Approaches to Problem Solving Survey (AAPS) was developed to measure students’ attitudes and approaches problem solving in physics. The format includes 33 multiple choice and agree/disagree items to be administered before and after the learning experience for a pre/post‐test comparison to quantify changes in student thinking. Each question is scored as +1 for alignment with expert‐thinking, ‐1 for disagreement with expert‐like thinking, and 0 for neutral responses. The total score for each student is calculated as a percentage of points of the total number possible, and the percent score on the survey was averaged for all students in the cohort to represent an aggregate pre‐test and post‐test score for the group.^27^

### 4.2 Colorado Learning Attitudes about Science Survey for Experimental Physics

To investigate student perceptions of gaps between classroom laboratory instruction and professional research, the Colorado Learning Attitudes about Science Survey (eCLASS) for Experimental Physics was administered to the students before and after the program.^28^ The survey contains 30 questions, and each response is scored as +1 for alignment, 0 for neutral, and ‐1 for disagreement with expert‐like responses. The total score for each student was calculated as a percentage of points of the total number possible, and the percent score of the survey was averaged for all students in the cohort to represent an aggregate pre‐test and post‐test score for the group.

### 4.3 Undergraduate Research Student Self‐Assessment

The Undergraduate Research Student Self‐Assessment (URSSA) is a survey instrument for assigning student outcomes of undergraduate research experiences measuring self‐reported gains in skill development, conceptual knowledge, understanding of the practical work of science, development of identity as a scientist, and clarity for future goals.^29^ Unlike a pre‐ and post‐test design where educators can calculate the difference between two different time points, this instrument is designed to be a reflection for student self‐assessment that occurs at the end of the learning experience. Highest scores are related to the most positive response for each category and the lowest scores correspond to the most negative responses. For example, responses are on scales of satisfaction (very satisfied, somewhat satisfied, somewhat dissatisfied, very dissatisfied, I did not do this), self‐reported gain or quantification of magnitude (great, good, a little, no(ne), I did not do this), ratings (excellent, good, fair, poor, not applicable), agreement (strongly agree, agree, disagree, strongly disagree), likelihood (much more, somewhat more, somewhat less, much less, not applicable), or binary response (yes/no). For survey items that reported self‐reported gains, the Likert responses were converted to numerical values on a 4‐point scale where 4 is the highest score. If “not applicable” or “I did not do this” were provided as a response to an item, the standard deviation was calculated from remaining responses.

### 4.4 Program evaluation data

Students in this summer program completed three surveys including the AAPS,^27^ Colorado eCLASS^28^ for Experimental Physics, and URSSA.^29^ The AAPS and eCLASS are designed to be conducted at the beginning and the end of the learning experience to compare changes in scores. The URSSA is designed to be administered at the completion of the program to evaluate attitudes and beliefs about science correlated to the program. The format of these assessments is mostly composed of Likert scales questions and open response for both qualitative and quantitative analysis with all of the questions shown for reference in the Supporting Information.

For assessment of pre‐ and post‐test scores, the average gain was calculated as the difference in attitudes before and after the program and a normalized gain, or Hake gain, as a measure of the effectiveness of the learning experience to compare what the students learned compared to what they could have learned as a way of accounting for previous experience shown in Equation (1).^30^

(1) $$\langle g\rangle =\frac{\langle post\rangle -\;\langle pre\rangle }{100\;-\;\langle pre\rangle }\;$$

## 5 RESULTS

The model for the undergraduate Team Science program for medical physics was piloted in the summer of 2024 with four undergraduate students. This group included students who self‐identified as being a part of historically marginalized groups including gender minorities, individuals with physical disabilities, and non‐native English speakers. Funding was provided by AAPM DREAM Fellowship Program, The Ohio State University Physics Department, Ohio University's Honors Tutorial College, and The Ohio State University Office of Academic Affairs—Undergraduate Research Access Innovation Seed Grant for supporting the students and the program.

The students collaborated to complete four distinct 2‐week projects throughout the summer. Every 2 weeks the team of students had new unique problems to solve, investigating different aspects of medical physics. Examples of projects included the design and characterization of a novel collimator to be used with ultra‐high dose rate (FLASH) radiation therapy that was used for a large animal study during the summer, the optimization of on‐board imaging protocols for a new linac imaging panel evaluating reconstruction algorithms, and the quantification of superficial vasculature using near‐infrared imaging. The final questions involved exploring how medical images could be made accessible to a person with visual impairment and whether a person with visual impairment could become board certified in medical physics.

### 5.1 Survey results

The students completed the beginning of the program surveys (AAPS and eCLASS) during the first week of the program and end of program surveys during the last week of the program (AAPS, eCLASS, and URSSA) through an online form. Results for the average scores and standard deviation of the AAPS and eCLASS assessments including gains from the summer experience in aggregate from the students are shown in Table 2.

**TABLE 2:** Results from pre‐ and post‐tests from students participating in pilot Team Science Research group for Attitudes and Approaches to Problem Solving Survey (AAPS) to measure students’ attitudes to problem solving and Colorado Learning Attitudes about Science Survey for Experimental Physics (eCLASS) to assess the students’ perceptions to the gap between classroom laboratory instruction and professional research.

Attitudes and Approaches to Problem Solving Survey (AAPS) *N* = 4			Colorado Learning Attitudes about Science Survey for Experimental Physics (eCLASS) *N* = 4		
	Average	STDEV		Average	STDEV
Pre‐test	78.8%	3.74%	Pre‐test	70.0%	2.94%
Post‐test	88.6%	0.96%	Post‐test	84.2%	0.50%
Gain	9.8%		Gain	14.2%	
Hake Gain	46.2%		Hake Gain	47.3%	

After reviewing the AAPS assessments, students on average gained 10% in scientific attitudes and approaches to problem solving showing more alignment with attitudes from experts in terms of problem solving and approaches over the 10‐week period of the program. Students in the program started at 79% and increased to 89%. As a benchmark, it has been reported that physics faculty on average have scored between 88%–92%, graduate physics students 62–73%, and introductory physics students 33%^27^ indicating students already had a high degree of expert thinking at the beginning of the program and this increased to scores approaching that of physics faculty by the end of the program. Gains were also demonstrated based on the eCLASS assessment with a 14% average gain in attitudes related to experimental physics after the program. The Hake Gain for both assessments is 46–47% which can be interpreted as how much gain the students were capable of increasing their physics expert‐like thinking during the program based on their baseline scores. Average cohort responses to the AAPS and eCLASS assessments can be found in Supporting Information for reference.

The students also independently completed the validated URSSA Survey anonymously via an online form at the end of the program. For survey items that reported self‐reported gains, the Likert responses were converted to numerical values on a 4‐point scale where 4 is the highest score. Table 3 shows categories with highest self‐reported gains including understanding what everyday research work is like, engaging in real‐world science research, and preparation for the future. While many of the scores also scored highly, areas recognized as weaknesses (< 3) from the standardized assessment have been identified including keeping a detailed lab notebook, working extra hours because you were excited about the research, housing, ethics seminars, training in human or animal subjects’ regulations, and interest in pursuing careers outside of STEM. A full report of student responses from self‐reported gains and binary reposes (yes/no) to outcomes from student experiences can be found in Supporting Information for reference.

**TABLE 3:** Summary of areas of value from pilot Team Science undergraduate program based on item responses to the Undergraduate Research Student Self‐Assessment (URSSA) (*N* = 4).

Items with average score: 4/4	Items with average score: 3.75/4
Understanding the theory and concepts guiding my research project	Figuring out the next step in a research project
Identifying limitations of research methods and designs	Understanding the relevance of research to my coursework
Understanding what everyday research work is like	Formulating a research question that could be answered with data
Engage in real‐world science research	Comfort in working collaboratively with others
My experience has prepared me for advanced course work or thesis work	Writing scientific reports or papers
My research experience has prepared me for a job	Try out new ideas or procedures on your own
My research has prepared me for graduate school	Think creatively about the project
My working relationship with my research mentor	Feel responsible for the project
The research experience overall	Managing my time
	Feel a part of the scientific community

Open response comments about the research experience were positive and are included in Table 4 summarizing the value of the team science summer research experience.

**TABLE 4:** Examples of open‐response comments from Team‐Science undergraduate program responses from the participants (*N* = 4).

Deidentified student feedback
“This research experience was really well structured, and I got a lot out of it. The hard part about undergraduate research is you don't usually get a lot of independent work to do. I sometimes like to think of being the "coffee person" as an undergrad in a research lab doing all of the tedious tasks that the master's and PhD students don't want to do. I never felt like that this summer, and I really felt that I was able to contribute my own thoughts to the research. I encouraged me to think like a scientist and understand what it is like to do research in the field. I would rate this experience a 10/10 and am very thankful for this opportunity.”
“The research that I was doing felt like it was actually significant in the field, and I could easily connect it to real world applications, so my work was very engaging and rewarding. I also felt like I was contributing a lot to the research being done and didn't feel like I was just an assistant.”
“I have determined that the research work in this field is something I definitely want to pursue. Thus, I will be applying to PhD programs and currently plan to pursue that as far as I can go.”
“This experience was incredibly formative for my graduate school plans and future career… I also learned that I love doing research, and that elements of it are possible for someone with low vision… I would never have learned all this if I hadn't come here. While parts of this summer were really challenging practically and emotionally, I am so glad that I learned what I did; I feel much more prepared to face the rest of my undergrad and start thinking about a career.”

### 5.2 Student professional development

Students were encouraged to present their research at their local institutions after the program, submit abstracts to related conferences, and\ publish manuscripts to disseminate their work. Examples include presenting proffered abstracts at the annual national American Association of Physicists in Medicine (AAPM) meeting with an oral presentation, poster discussion, and virtual posters, presentations at regional chapter meetings, a joint abstract accepted for an international conference. Other studies are still on‐going which will lead to submissions of manuscripts to peer reviewed journals. Opportunities for in person conference attendance was strongly encouraged to continue to build their networks within the field.

## 6 DISCUSSION

The pilot year provided valuable feedback for applying a Team Science approach in medical physics, establishing feasibility of the model, providing experience, and generating data for refining the program for mentoring multiple undergraduate students. The goal of disseminating information about this program's structure is to provide an alternative and sustainable way for expanding medical physics research opportunities. This program was piloted with four students but theorize that the method can be expanded to larger groups of students where tools, like the StrengthsFinder assessment, can be used to help inform the development of multiple teams within a larger program.

The program started with the first two research question cycles as fairly regimented, facilitating a framework for focused efforts toward the specific question questions with the new team. Once students were more comfortable working together and gained confidence in their research skills, individuals who wanted to gain deeper understanding of topics from previous questions had the opportunity to continue to build toward more concrete academic products while still contributing to work toward answering the new research questions. This allowed students to tailor their learning to their interests and use their strengths, providing a degree of autonomy within the structured program for participants. While the four roles were assigned to each project formally at the beginning of the cycle, the roles became more fluid as the individuals became a highly functioning team where students were able to identify their own strengths and those of their team members to optimize and harmonize achievement of the team goals.

Validated assessments were used in this program review to provide quantitative data for student gains in attitudes through the program. While assessments have been used in physics undergraduate education, this is the first known application for assessments in undergraduate research experiences in medical physics. As educators continue to mentor students, it is encouraged that standardized assessments be considered to help benchmark educational strategies to understand how to be help learners reach their goals.

While the pilot program was primarily focused on the research questions and project management, area for improvement were highlighted in the URSSA assessment including the integration of professional and ethical discussions throughout the program and keeping a detailed lab notebook. One strategy for inclusion is collaboration with the university's summer undergraduate research consortium and the topics can be highlighted and modeled throughout the week by the program director and research mentors. Incorporating responsible research conduct training during the program's onboarding week ensures that all participants are equipped with a foundational understanding prior to initiating any research activities.

Throughout the summer, the program director provided feedback verbally in both group and individual settings. In addition to verbal feedback, communication about progress could be more formalized by a written evaluation or review. Individual learners could be provided written feedback from mentors which is structured in a rubric for each cycle or provided feedback for their peers curated by the program director for each research question cycle. Examples of written feedback would be specific to the program and rotation goals and could be adapted from existing literature for undergraduate research.^31^, ^32^, ^33^

Building a strong team environment is key for success of the program. Developing trust with members of the team is crucial for engagement with the research questions and facilitating healthy group dynamics. One aspect that was not standardized in the pilot program was the start and end dates of the participants based on non‐uniform funding sources for individual students participating in the pilot. Ideally this would be synchronized amongst all the students so that program expectations could be standardized for onboarding, team building, and continuity of expectations for outcomes. Creating more organized social events, providing student housing together on campus, and creating opportunities for collaboration outside of work are ways that the program could be improved to continue to build relationships, especially for students without preexisting regional social support.

## 7 CONCLUSION

Hosting a team of undergraduate research students was a valuable educational experience for students and faculty members within our medical physics group. Using a Team Science approach allowed for students to explore multiple questions during the summer and experience a robust and integrated approach to how research is conducted in the field of medical physics. We look to expand and adapt this framework in the future so more students will have the opportunity to participate in a positive learning experience when considering medical physics as a vocation for applying physics knowledge to advance human health.

## CONFLICT OF INTEREST STATEMENT

Student funding was provided by The Ohio State University Office of Academic Affairs Undergraduate Research Access Innovation Seed Grant, The Ohio State University Physics Department Summer Research Program, the AAPM DREAM Fellowship Program, and the Ohio University's Honors Tutorial College.

## Supporting information



Supporting Information

## Data Availability

The data that support the findings of this study are available from the corresponding author, AJC, upon reasonable request.
