# Supplementary material for: Implementing team science in undergraduate medical physics research
Source: J Appl Clin Med Phys. 2025 Jul 14;26(7):e70169. doi: 10.1002/acm2.70169 (PMC12257336; doi:10.1002/acm2.70169)
Supplement: Supplementary file 1 — Supporting Information [file ACM2-26-e70169-s001.docx]

# Supplementary Materials

## AAPS Data

The Attitudes and Approaches to Problem Solving Survey (AAPS) was developed to measure students’ attitudes and approaches problem solving in physics. **Table S1** includes average responses from the student cohort for each item compared to expert responses with each question scored as +1 for alignment, 0 for neutral, and -1 for disagreement with expert-like responses at the beginning and the end of the program.

Table S1: Averaged student data from AAPS surveys before and after the summer research program.

| **Attitudes and Approaches to Problem Solving Survey (AAPS)** | **Pre-Test Average** | **Pre-Test STDEV** | **Post-Test Average** | **Post-Test STDEV** |
| --- | --- | --- | --- | --- |
| If I’m not sure about the right way to start a problem, I’m stuck unless I go see the teacher/TA or someone else for help. | -0.3 | 1.0 | 1.0 | 0.0 |
| When solving physics problems, I often make approximations about the physical world. | 1.0 | 0.0 | 1.0 | 0.0 |
| In solving problems in physics, being able to handle the mathematics is the most important part of the process. | 1.0 | 0.0 | 0.8 | 0.5 |
| In solving problems in physics, I always identify the physics principles involved in the problem first before looking for corresponding equations. | 1.0 | 0.0 | 1.0 | 0.0 |
| “Problem solving” in physics basically means matching problems with the correct equations and then substituting values to get a number. | 0.5 | 1.0 | 0.8 | 0.5 |
| In solving problems in physics, I can often tell when my work and/or answer is wrong, even without looking at the answer in the back of the book or talking to someone else about it. | 1.0 | 0.0 | 1.0 | 0.0 |
| To be able to use an equation to solve a problem (particularly in a problem that I haven’t seen before), I think about what each term in the equation represents and how it matches the problem situation. | 0.5 | 1.0 | 0.8 | 0.5 |
| There is usually only one correct way to solve a given problem in physics. | 1.0 | 0.0 | 1.0 | 0.0 |
| I use a similar approach to solving all problems in volving conservation of linear momentum even if the physics situations given in the problems are very different. | 0.8 | 0.5 | 1.0 | 0.0 |
| If I am not sure about the correct approach to solving a problem, I will reflect upon physics principles that may apply and see if they yield a reasonable solution. | 1.0 | 0.0 | 1.0 | 0.0 |
| Equations are not things that one needs to understand in an intuitive sense; I routinely use equations to calculate numerical answers even if they are non-intuitive. | 1.0 | 0.0 | 0.5 | 1.0 |
| Physics involves many equations each of which applies primarily to a specific situation. | -1.0 | 0.0 | 0.8 | 0.5 |
| If I used two different approaches to solve a physics problem and they gave different answers, I would spend considerable time thinking about which approach is more reasonable. | 1.0 | 0.0 | 1.0 | 0.0 |
| When I solve physics problems, I always explicitly think about the concepts that underlie the problem. | 1.0 | 0.0 | 1.0 | 0.0 |
| When solving physics problems, I often find it useful to first draw a picture or a diagram of the situations described in the problems. | 1.0 | 0.0 | 1.0 | 0.0 |
| When answering conceptual physics questions, I mostly use my “gut” feeling rather than using the physics principles I usually think about when solving quantitative problems. | 0.0 | 1.2 | 0.0 | 1.2 |
| I am equally likely to draw pictures and/or diagrams when answering a multiple-choice question or a corresponding free-response (essay) question. | 1.0 | 0.0 | 0.8 | 0.5 |
| I usually draw pictures and/or diagrams even if there is no partial credit for drawing them. | 1.0 | 0.0 | 0.8 | 0.5 |
| I am equally likely to do scratch work when answering a multiple-choice question or a corresponding free-response (essay) question. | 1.0 | 0.0 | 1.0 | 0.0 |
| After I solve each physics homework problem, I take the time to reflect on and learn from the problem solution. | 0.5 | 1.0 | 0.8 | 0.5 |
| After I have solved several physics problems in which the same principle is applied in different context, I should be able to apply the same principle in other situations. | 1.0 | 0.0 | 1.0 | 0.0 |
| If I obtain an answer to a physics problem that does not seem reasonable, I spend considerable time thinking about what may be wrong with the problem solution. | 1.0 | 0.0 | 1.0 | 0.0 |
| If I cannot solve a physics problem in 10 minutes, I give up on that problem. | 0.8 | 0.5 | 1.0 | 0.0 |
| When I have difficulty solving a physics homework problem, I like to make sure that I learn from my mistakes and do not make the same mistakes again. | 0.8 | 0.5 | 1.0 | 0.0 |
| When I do not get a question correct on a test or homework, I always make sure I learn from my mistakes and do not make the same mistakes again. | 1.0 | 0.0 | 1.0 | 0.0 |
| It is more useful for me to solve a few difficult problems using a systematic approach and learn from them rather than solving many similar easy problems one after another. | 0.8 | 0.5 | 1.0 | 0.0 |
| I enjoy solving physics problems even though it can be challenging at times. | 1.0 | 0.0 | 1.0 | 0.0 |
| I try different approaches if one approach does not work. | 1.0 | 0.0 | 1.0 | 0.0 |
| If I realize that my answer to a physics problem is not reasonable, I trace back my solution to see where I went wrong. | 1.0 | 0.0 | 1.0 | 0.0 |
| It is much more difficult to solve a physics problem with symbols than solving an identical problem with a numerical answer. | 0.8 | 0.5 | 1.0 | 0.0 |
| While solving a physics problem with a numerical answer, I prefer to solve the problem symbolically first and only plug in the numbers at the very end. | 1.0 | 0.0 | 1.0 | 0.0 |
| Suppose you are given two problems. One problem is about a block sliding down an inclined plane with no friction present. The other problem is about a person swinging on a rope. Air resistance is negligible. You are told that bother problems can be solved using the concept of conservation of mechanical energy of the system. Which one of the following statements do you MOST agree with? | 1.0 | 0.0 | 1.0 | 0.0 |
| Suppose you are given two problems. One problem is about a block sliding down an included plane. There is friction between the block and the incline. The other problem is about a person swinging on a rope. There is air resistance between the person and air molecules. You are told that both problems can be solved using the concept of conservation of total (not just mechanical) energy. Which of the following statements do you MOST agree with? | 1.0 | 0.0 | 0.5 | 1.0 |

## eCLASS Data

The Colorado Learning Attitudes about Science Survey (CLASS) for Experimental Physics was developed to investigate student perceptions of gaps between classroom laboratory instruction and professional research. It was administered to the students before and after the program**. Table S2** includes average responses from the student cohort for each item compared to expert responses with each question scored as +1 for alignment, 0 for neutral, and -1 for disagreement with expert-like responses at the beginning and the end of the program.

Table S2: Averaged student data from eCLASS surveys before and after the summer research program

| **Colorado Learning Attitudes about Science Survey (CLASS) for Experimental Physics** | **Pre-Test Average** | **Pre-Test STDEV** | **Post-Test Average** | **Post-Test STDEV** |
| --- | --- | --- | --- | --- |
| When doing an experiment, I try to understand how the experimental setup works. | 1.0 | 0.0 | 1.0 | 0.0 |
| If I wanted to, I think I could be good at doing research. | 0.8 | 0.5 | 1.0 | 0.0 |
| When doing a physics experiment, I don't think much about sources of systematic error. | 0.5 | 0.6 | 0.8 | 0.5 |
| If I am communicating results from an experiment, my main goal is to have the correct sections and formatting. | 0.0 | 0.8 | 0.3 | 1.0 |
| Calculating uncertainties usually helps me understand my results better. | -0.8 | 0.5 | -0.8 | 0.5 |
| Scientific journal articles are helpful for answering my own questions and designing experiments | 0.5 | 1.0 | 1.0 | 0.0 |
| I don't enjoy doing physics experiments. | 1.0 | 0.0 | 1.0 | 0.0 |
| When doing an experiment, I try to understand the relevant equations. | 1.0 | 0.0 | 1.0 | 0.0 |
| When I approach a new piece of lab equipment, I feel confident I can learn how to use it well enough for my purposes. | 0.5 | 1.0 | 0.8 | 0.5 |
| Whenever I use a new measurement tool, I try to understand its performance limitations. | 1.0 | 0.0 | 1.0 | 0.0 |
| Computers are helpful for plotting and analyzing data. | 1.0 | 0.0 | 1.0 | 0.0 |
| I don't need to understand how the measurement tools and sensors work in order to carry out an experiment. | 0.8 | 0.5 | 1.0 | 0.0 |
| If I try hard enough, I can succeed at doing physics experiments. | 1.0 | 0.0 | 1.0 | 0.0 |
| When doing an experiment, I usually think up my own questions to investigate. | 0.8 | 0.5 | 1.0 | 0.0 |
| Designing and building things is an important part of doing physics experiments. | 0.5 | 1.0 | 1.0 | 0.0 |
| The primary purpose of doing a physics experiment is to confirm previously known results. | 0.5 | 1.0 | 1.0 | 0.0 |
| When I encounter difficulties in the lab, my first step is to ask an expert, like the instructor. | 0.3 | 1.0 | 0.5 | 0.6 |
| Communicating scientific results to peers is a valuable part of doing physics experiments. | 1.0 | 0.0 | 1.0 | 0.0 |
| Working in a group is an important part of doing physics experiments. | 1.0 | 0.0 | 1.0 | 0.0 |
| I enjoy building things and working with my hands. | 1.0 | 0.0 | 1.0 | 0.0 |
| I am usually able to complete an experiment without understanding the equations and physics ideas that describe the system I am investigating. | 1.0 | 0.0 | 0.8 | 0.5 |
| If I am communicating results from an experiment, my main goal is to make conclusions based on my data using scientific reasoning. | 1.0 | 0.0 | 1.0 | 0.0 |
| When I am doing an experiment, I try to make predictions to see if my results are reasonable. | 1.0 | 0.0 | 1.0 | 0.0 |
| Nearly all students are capable of doing a physics experiment if they work at it. | 1.0 | 0.0 | 1.0 | 0.0 |
| A common approach for fixing a problem with an experiment is to randomly change things until the problem goes away. | -0.3 | 1.0 | 1.0 | 0.0 |
| It is helpful to understand the assumptions that go into making predictions. | 1.0 | 0.0 | 1.0 | 0.0 |
| When doing an experiment, I just follow the instructions without thinking about their purpose. | 1.0 | 0.0 | 1.0 | 0.0 |
| I do not expect doing an experiment to help my understanding of physics. | 1.0 | 0.0 | 1.0 | 0.0 |
| If I don't have clear directions for analyzing data, I am not sure how to choose an appropriate analysis method. | 0.0 | 1.2 | 0.0 | 0.8 |
| Physics experiments contribute to the growth of scientific knowledge | 1.0 | 0.0 | 1.0 | 0.0 |

## URSSA Data

The Undergraduate Research Student Self-Assessment (URSSA) is a survey instrument for assigning student outcomes of undergraduate research experiences measuring self-reported gains in skill development, conceptual knowledge, understanding of the practical work of science, development of identity as a scientist, and clarity for future goals. This instrument is designed to be a reflection for student self-assessment that occurs at the end of the learning experience. For survey items that reported self-reported gains, the Likert responses were converted to numerical values on a 4-point scale where 4 is the highest score shown in **Table S3**. If “not applicable” or “I did not do this” were provided as a response to an item, the standard deviation was calculated from remaining responses.

Table S3: Averaged student data from the URSSA at the end of the summer research program

| **How much did you gain in the following areas as a result of your most recent research experience?** | **Average Score (1-4)** | **STDEV** |
| --- | --- | --- |
| Analyzing data for patterns | 3.3 | 1.0 |
| Figuring out the next step in a research project | 3.8 | 0.5 |
| Understanding the theory and concepts guiding my research project | 4.0 | 0.0 |
| Understanding the connections among scientific disciplines | 3.5 | 1.0 |
| Understanding the relevance of research to my coursework | 3.8 | 0.5 |
| Formulating a research question that could be answered with data | 3.8 | 0.5 |
| Identifying limitations of research methods and designs | 4.0 | 0.0 |
| Confidence in my ability to contribute to science | 3.3 | 1.0 |
| Comfort in discussing scientific concepts with others | 3.3 | 0.5 |
| Comfort in working collaboratively with others | 3.8 | 0.5 |
| Confidence in my ability to do well in future science courses | 3.5 | 0.6 |
| Ability to work independently | 3.0 | 0.8 |
| Developing patience with the slow pace of research | 3.0 | 1.2 |
| Understanding what everyday research work is like | 4.0 | 0.0 |
| Taking greater care in conducting procedures in the lab or field | 3.3 | 1.0 |
| Writing scientific reports or papers | 3.8 | 0.5 |
| Making oral presentations | 3.0 | 1.2 |
| Defending an argument when asked questions | 3.0 | 0.8 |
| Explaining my project to people outside my field | 3.3 | 1.0 |
| Preparing a scientific poster* | 3.0 | 1.7 |
| Keeping a detailed lab notebook | 2.8 | 1.0 |
| Conducting observations in the lab or field | 3.5 | 0.6 |
| Using statistics to analyze data* | 3.3 | 0.6 |
| Calibrating instruments needed for measurement* | 3.7 | 0.6 |
| Working with computers | 3.3 | 1.0 |
| Understanding journal articles | 3.0 | 0.0 |
| Conducting database or internet searches | 3.0 | 0.8 |
| Managing my time | 3.8 | 0.5 |

*indicates one of the respondents indicated “not applicable” to this category

| **During your research experience how much did you…** | **Average Score (1-4)** | **STDEV** |
| --- | --- | --- |
| Engage in real-world science research | 4.0 | 0.0 |
| Feel like a scientist | 3.8 | 0.5 |
| Think creatively about the project | 3.8 | 0.5 |
| Try out new ideas or procedures on your own | 3.8 | 0.5 |
| Feel responsible for the project | 3.8 | 0.5 |
| Work extra hours because you were excited about the research | 2.8 | 1.0 |
| Interact with scientists from outside your school | 3.3 | 1.0 |
| Feel a part of a scientific community | 3.8 | 0.5 |

|  |  |  |
| --- | --- | --- |
|  |  |  |
|  |  |  |
|  |  |  |
|  |  |  |
|  |  |  |
|  |  |  |
|  |  |  |
|  |  |  |

| **Rate how much you agree with the following statements** | **Average Score (1-4)** | **STDEV** |
| --- | --- | --- |
| Doing research confirmed my interest in my field of study. | 3.5 | 0.6 |
| Doing research clarified for me which field of study I want to pursue. | 3.5 | 0.6 |
| My research experience has prepared me for advanced coursework or thesis work. | 4.0 | 0.0 |
| My research experience has prepared me for graduate school. | 4.0 | 0.0 |
| My research experience has prepared me for a job. | 4.0 | 0.0 |

| **Compared to your intentions before doing research, how likely are you now to:** | **Average Score (1-4)** | **STDEV** |
| --- | --- | --- |
| ...enroll in a Ph.D. program in science, mathematics or engineering? | 3.8 | 0.5 |
| ...enroll in a masters program in science, mathematics or engineering? | 3.0 | 1.2 |
| ...enroll in a combined M.D./Ph.D. program?** | 2.5 | 0.7 |
| ...enroll in a program to earn a different professional degree (i.e., in law, veterinary medicine, etc.)?* | 1.7 | 1.2 |
| ...enroll in medical or dental school?* | 2.0 | 1.7 |
| …pursue certification as a teacher?* | 2.3 | 1.2 |
| ...work in a science lab | 3.5 | 0.6 |

*indicates one of the respondents indicated “not applicable” to this category

**indicates two of the respondents indicated “not applicable” to this category

| **The following questions ask about formal program activities that may have been offered as part of your research experience.** | **Average Score (1-4)** | **STDEV** |
| --- | --- | --- |
| The application process** | 3.0 | 0.0 |
| Information available to help me choose a research project | 3.8 | 0.5 |
| Ease in locating a research mentor* | 4.0 | 0.0 |
| Ease in securing a research position* | 3.7 | 0.6 |
| Lab or field equipment | 4.0 | 0.0 |
| Support and guidance from program staff | 3.8 | 0.5 |
| Support and guidance from my research mentor | 4.0 | 0.0 |
| Support and guidance from other research group members | 4.0 | 0.0 |
| Research group meetings | 4.0 | 0.0 |
| Financial support** | 4.0 | 0.0 |
| Housing*** | 1.0 | 0.0 |
| Field trips | 4.0 | 0.0 |
| Group social activities | 3.5 | 1.0 |
| Session(s) offered on graduate school programs or careers | 4.0 | 0.0 |
| Information provided by the career counseling center | 3.0 | 0.0 |
| Field trips*** | 3.5 | 0.7 |
| Workshop(s) on science writing and presentation | 3.0 | 0.0 |
| Training in library/internet/database search methods | 3.0 | 1.0 |
| Safety training | 3.3 | 1.0 |
| Ethics seminar(s) | 2.7 | 0.6 |
| Training in human or animal subjects regulations | 2.8 | 1.0 |

*indicates one of the respondents indicated “not applicable” to this category

**indicates two of the respondents indicated “not applicable” to this category
***indicates three of the respondents indicated “not applicable” to this category

| **As part of my most recent research experience…** | **Average Score (1=yes, 0=no)** | **STDEV** |
| --- | --- | --- |
| I presented a talk or poster to other students and faculty | 0.8 | 0.5 |
| I will present a talk or poster to other students and faculty | 1.0 | 0.0 |
| I presented a talk or poster at a professional conference | 0.3 | 0.5 |
| I will present a talk or poster at a professional conference | 0.3 | 0.5 |
| I attended a conference | 0.5 | 0.6 |
| I will attend a conference | 0.3 | 0.5 |
| I wrote or co-wrote a paper that was published in an academic journal | 0.0 | 0.0 |
| I will write or co-write a paper to be published in an academic journal | 1.0 | 0.0 |
| I wrote or co-write a paper to be published in an undergraduate research journal | 0.0 | 0.0 |
| I will write or co-write a paper to be published in an undergraduate research journal | 0.5 | 0.6 |
| I won an award or scholarship based on my research | 0.0 | 0.0 |
